# Research progress on the mechanism of TCM regulating intestinal microbiota in the treatment of DM mellitus

**DOI:** 10.3389/fendo.2024.1308016

**Published:** 2024-03-27

**Authors:** Yang Ping, Jianing Liu, Lihong Wang, Hongbin Qiu, Yu Zhang

**Affiliations:** ^1^ College of Pharmacy, Jiamusi University, Jiamusi, Heilongjiang, China; ^2^ Heilongjiang Pharmaceutical Research Institute, Jiamusi, Heilongjiang, China

**Keywords:** traditional Chinese medicine, diabetes mellitus, intestinal microbiota, disorder, mechanism

## Abstract

In recent years, with the improvement of people’s living standards, the incidence of DM has increased year by year in China. DM is a common metabolic syndrome characterized by hyperglycemia caused by genetic, environmental and other factors. At the same time, long-term suffering from DM will also have an impact on the heart, blood vessels, eyes, kidneys and nerves, and associated serious diseases. The human body has a large and complex gut microbiota, which has a significant impact on the body’s metabolism. Research shows that the occurrence and development of DM and its complications are closely related to intestinal microbiota. At present, western medicine generally treats DM with drugs. The hypoglycemic effect is fast and strong, but it can have a series of side effects on the human body. Compared with western medicine, Chinese medicine has its unique views and methods in treating DM. TCM can improve symptoms and treat complications by improving the imbalance of microbiota in patients with DM. Its characteristics of health, safety, and reliability are widely accepted by the general public. This article reviews the relationship between intestinal microbiota and DM, as well as the mechanism of TCM intervention in DM by regulating intestinal microbiota.

## 1 Introduction

Diabetes mellitus (DM) is a common metabolic syndrome characterized by hyperglycemia caused by genetic, environmental and other factors, and has become a major medical and health problem threatening global human health (1). In 2017, the eighth edition of the International DM Federation (IDF) DM Atlas showed that there were about 425 million DM patients worldwide. It is estimated that by 2045, the number of patients with DM will increase to 700 million (2). The incidence rate of DM in China is about 10% (3), and the number of DM patients has reached 114 million, accounting for 1/3 of the total number of DM patients in the world. The pathological process of DM is very complex, and its pathogenesis has not been fully explored. The influencing factors include genetic factors, related cytokines, diet, environment, early colonization of intestinal microbiota, breastfeeding, intestinal proinsulin effect, delivery mode, and other factors (4). The initial clinical manifestations of DM are polydipsia, polydipsia, polyuria and weight loss (5). However, in the long run, it can have an impact on the heart, blood vessels, eyes, kidneys, and nerves, leading to serious related diseases (6). Such as DM heart disease, DM ketoacidosis, DM retinopathy, DM nephropathy, DM foot and other critical complications. This seriously affects human health and increases social burden (7). At present, western medicine generally treats DM with drugs. For example, stimulating insulin secretion and increasing its biological activity, reducing the absorption rate of glucose in the intestine. And increase the utilization of glucose by tissues in the body and promote the synthesis of liver glycogen, or directly inject exogenous insulin. The hypoglycemic effect is fast and strong, but it can produce a series of side effects. For example, patients have poor compliance and are prone to dependency. As the duration of illness prolongs, the dosage and types of hypoglycemic drugs taken continue to increase (8), with significant toxic side effects (9). And its pathway of action is single, and some Western medicines are prone to damage to the heart, stomach, liver, and kidneys (7). Therefore, it is particularly important to pay attention to the occurrence of DM and look for ways to intervene in DM and its complications.

Early colonization of gut microbiota begins at birth, and strains from the mother’s skin or vagina only briefly colonize within the baby’s body. The gut microbiota of 2-3 year old children is similar to that of adults. The diversity of gut microbiota in the population aged 60-70 decreases (10). The gut microbiota is a complex ecosystem composed of microbial communities, including trillions of bacteria, spanning at least 1000 different species. The gut microbiota is mainly composed of bacteria, but also contains other symbiotes such as archaea, viruses, fungi, and protists (11). It includes probiotics that can ensure the health of the body and inhibit the proliferation of pathogenic bacteria, as well as neutral bacteria that may have pathogenic effects under certain factors. The gut microbiota is considered to play an important role in enhancing host immunity, promoting food digestion, regulating intestinal endocrine function, regulating neural signals, regulating drug action, regulating metabolism, detoxification, and other aspects (12). In a healthy state, the gut microbiota coexists harmoniously with the host in a relatively stable dynamic equilibrium environment, and symbiotic microorganisms benefit from the abundant nutrients provided by the gut to survive. The numerous metabolites produced by intestinal microorganisms also have key functions in regulating the host, such as maintaining energy homeostasis, inhibiting the invasion and reproduction of intestinal pathogenic microorganisms, etc. (10, 13). Once this balance is disrupted, the types, quantities, proportions, and positions of gut bacteria will become disrupted, leading to ecological imbalance (14). The imbalance of gut microbiota is closely related to the health of the body. There are many factors that can induce gut microbiota imbalance, such as living environment, genetics, age, gender, diet, and medication, all of which can affect the abundance and diversity of the human gut microbiota. Dysfunction of intestinal microbiota is also associated with many diseases, such as DM, Parkinson’s disease (PD), colorectal cancer, hepatocellular carcinoma, chronic kidney disease (CKD) and ischemic stroke. The imbalance of intestinal microbiota, the increase of intestinal mucosal permeability, the change of intestinal immune response and other factors will participate in the occurrence of type 1 DM(T1DM) (15, 16). And as blood sugar rises, the number of enterococci increases, while the number of bifidobacteria and common bifidobacteria decreases. It can promote the production of harmful substances in the intestines and worsen the condition of type 2 DM(T2DM) (17). Therefore, regulating the intestinal microbiota and improving the intestinal ecological imbalance are considered to be an important direction for the treatment of DM.

Traditional Chinese medicine(TCM) has obvious advantages in improving the symptoms of DM and treating complications, and its health, safety and reliability are accepted by the masses (18). DM has no corresponding disease name in TCM. We classify it as DM according to its symptom characteristics and the law of disease changes (9). TCM believes that the main causes of this disease include insufficient innate endowment, acquired dietary disorders, emotional disorders, and excessive labor and desire. The pathogenesis is based on yin deficiency and marked by dryness and heat, and the two are mutually causal and influential. The occurrence of DM is related to organs such as the lungs, stomach, and kidneys, and the three affect each other, ultimately leading to lung dryness, stomach heat, and kidney deficiency. Over time, DM can lead to damage to the yang qi and blood stasis, resulting in deficiency of both yin and yang, blood stasis, and various complications. Treatment should focus on nourishing yin and generating fluids, supplementing qi and kidney (19). In recent years, it has been found that intestinal microbiota and its metabolites participate in the prevention and treatment of DM from multiple pathways. Oral administration is the main mode of administration for TCM. After entering the intestine through the digestive tract, TCM inevitably comes into close contact with the gut microbiota and interacts with each other (20). Some TCM ingredients can regulate the composition and structure of gut microbiota, improve the gut microbiota, and further promote the transformation and utilization of bioactive ingredients in TCM (21). These active ingredients have an important impact on liver, intestine, stomach, pancreas, brain and other organs involved in sugar metabolism by regulating intestinal microbiota. It can be seen that there is a two-way relationship between intestinal microbiota and TCM, and intestinal microbiota is also a potential target for TCM to treat DM (22). This article will focus on the relationship between DM and its complications and intestinal microbiota, and clarify the relevant mechanism of Chinese medicine intervention in DM by regulating intestinal microbiota, in order to provide new ideas and research directions for DM and its complications.

## 2 DM and intestinal microbiota

### 2.1 Relationship between DM and intestinal microbiota imbalance

Diabetes is a metabolic disease, usually characterized by elevated blood glucose levels, which needs to be monitored and properly controlled (23). According to the Chinese Expert Consensus on DM Classification and Diagnosis released in February 2022, DM is divided into six types: type 1 DM (T1DM), type 2 DM (T2DM), monogenic DM, secondary DM, gestational DM (GDM) and unformed DM (24). But the most common types are type 1 and type 2 diabetes. Type 1 diabetes (T1DM) is usually associated with insulin production failure, which is due to t cell mediated autoimmune damage to the pancreas β Cells (25). On the other hand, type 2 diabetes (T2DM) is characterized by insulin resistance and decreased insulin secretion. Recent research shows that the appearance of intestinal microbiota plays a key role in the development of diabetes. The imbalance of intestinal microbiota, the increase of intestinal mucosal permeability, the change of intestinal immune response and other factors will participate in the occurrence of T1DM (26, 27). The change of intestinal microbiota can cause metabolic disorder of glucose, lipid, bile acid, etc., and produce inflammatory reaction, leading to functional disorder of the body. Insulin sensitivity and the utilization of sugar by peripheral tissues are reduced, thus leading to the occurrence of T2DM (28, 29).

Studies have shown that there is a significant correlation between the number of intestinal microbiota and the development of diabetes. A study on elderly patients with DM showed that the number of bifidobacteria and lactobacilli and the proportion of bifidobacteria/enterobacteriaceae (B/E) in elderly patients with DM significantly decreased, while enterococci and enterobacteriaceae significantly increased (30, 31). BIASSONI et al. (32) used 16sRNA sequencing technology to compare the gut microbiota of 31 children with T1DM and 25 healthy children. They found that the abundance of Bacteroidetes and Proteobacteria increased in T1DM patients, while the genera Desulfovibrio and Choledophilia increased beta the abundance of Proteobacteria decreased. DEMIRCI et al. (16) detected the levels of Bacteroidetes and Firmicutes in fecal samples from 53 T1DM patients. Compared with healthy individuals, T1DM patients had an increased level of Bacteroidetes in their gut microbiota, a decreased number of Firmicutes, and a significant decrease in the ratio of Firmicutes to Firmicutes. This indicates that there are changes in the abundance of gut microbiota in the human body before and after the onset of T1DM. He Yasha et al. (17) showed that T2DM mice induced by high-fat and high-sugar diet can have intestinal microbiota disorder. The intestinal microbiota rich in endotoxin in mice increased, and the level of lipopolysaccharide (LPS) in plasma increased, suggesting that intestinal microbiota disorder is related to diet induced DM.

**Figure 1:**
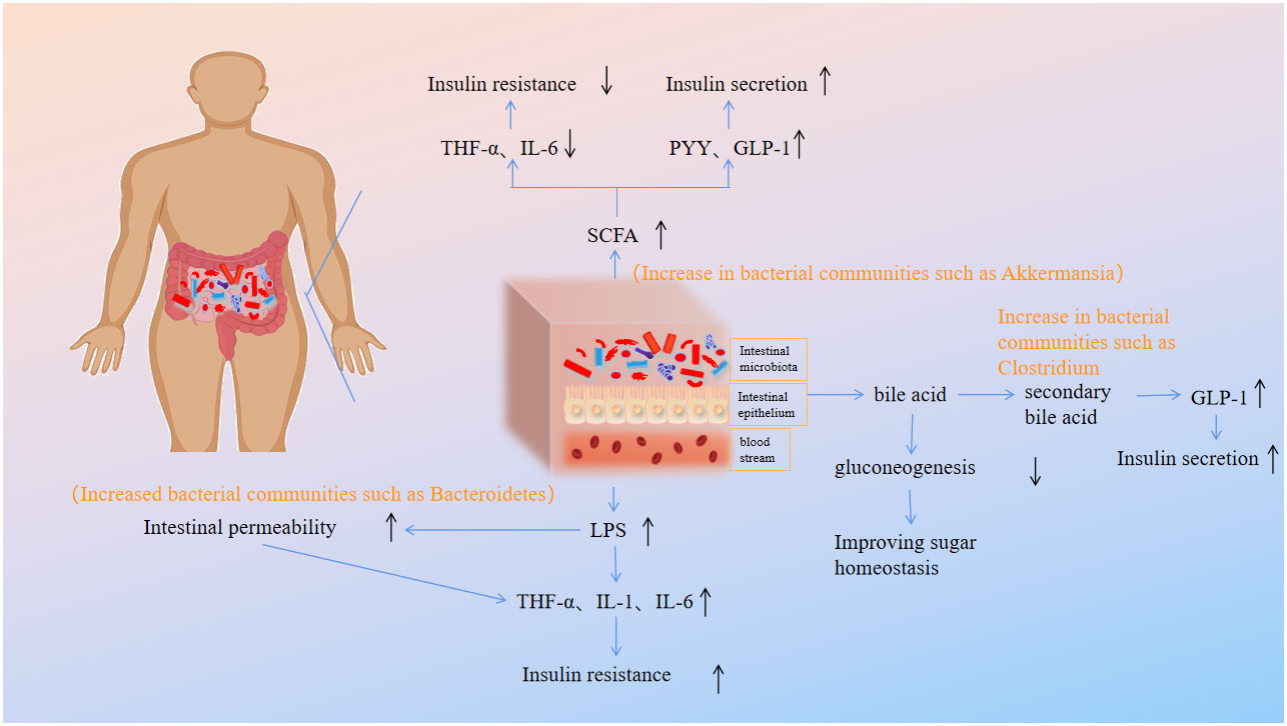
The mechanism of intestinal microbiota and DM.

In addition, there are also corresponding findings in the intestinal microbiota of patients with DM. Zeng Yipeng et al. (33) confirmed that the diversity of intestinal microbiota in patients with T2DM was significantly reduced, and the abundance and distribution of beneficial bacteria were significantly reduced, indicating that the metabolic level of the body was closely related to the intestinal microbiota. Li Li et al. (34) showed that probiotics can improve the blood sugar, blood lipid and oxidative stress water of newly overweight DM patients on average, indicating that intestinal microbiota has immune intervention effect, which can delay and prevent the occurrence and development of DM. Qin Junjie et al. (35) used the macro gene deep shotgun sequencing technology to detect and analyze the fecal microbiota of T2DM patients, and found that the abundance of beneficial bacteria in the intestinal tract, such as butyrate producing bacteria, decreased, while various opportunistic pathogens increased significantly. In the pathological state of DM, the number of gram-negative bacteria in the intestinal tract increases, and the excess endotoxin produced is released into the blood, causing endotoxemia, which causes systemic nonspecific inflammatory reaction, promotes insulin resistance, and further aggravates the abnormality of glucose metabolism (36). To sum up, the intestinal bacteria of DM patients will produce obvious changes in type and quantity before and after the onset of disease, and the development of DM can be intervened or improved by regulating the intestinal microbiota. The relationship between the occurrence and development of diabetes and the changes of intestinal flora is shown in **Figure 1**.

### 2.2 DM and its complications and intestinal microbiota

The microvascular complications related to small vessels in T2DM include DM nephropathy, DM retinopathy and DM neuropathy (37). A large number of studies have shown that there is a significant correlation between the changes in the composition of intestinal microbiota and the development of DM.

#### 2.2.1 DM nephropathy

DM nephropathy (DN) accounts for about 40% of poorly managed DM patients (38). It is the main microvascular complication of DM patients and the main cause of end stage renal disease (ESRD). Its main characteristics are a decrease in glomerular filtration rate and an increase in proteinuria. The intestine is the host of various microorganisms. Normal gut microbiota plays a profound role in the overall health of the host by participating in nutrient assimilation, metabolic processes, physiological functions, and immune responses. The gut kidney axis refers to the interaction between the gastrointestinal tract and the kidneys, and the gut microbiota is the core participant of this axis (39). Recent studies have shown that products of bacterial metabolism have been shown to affect the occurrence and progression of chronic kidney disease (40), leading to renal failure and worsening of gut microbiota imbalance (41). Kikuchi et al. found that the microbial metabolite phenyl sulfate (PS) increased in DM rats and DN patients, and its level was consistent with the patient’s basic proteinuria level. PS can cause podocyte injury and proteinuria in DM rats by inhibiting tyrosine phenolysis enzyme (tyrosine phenolysis enzyme is an enzyme that catalyzes the synthesis of phenol from tyrosine in diet to reduce proteinuria) (42). A study has found that streptozotocin induced DN mice were used to analyze the effect of microbiota on renal function. Divide the mice into two groups based on their urine protein levels. There are severe proteinuria (SP) mice and mild proteinuria (MP) mice. The composition of the two groups of bacteria is different. The abundance of Firmicutes in the SP group decreased, while the abundance of Firmicutes in the MP group increased. Compared with the MP group, Allobaculum was significantly enriched in the SP group. This leads to weight gain and elevated glucose levels, accelerating the evolution of DN (43). A study by Salguero et al. (44) revealed that there is an ecological imbalance in the gut microbiota of T2DM-CKD patients, including Proteobacteria, Verrucomicrobia, and Fusobacteria, which are Gram negative bacteria and are associated with elevated levels of LPS endotoxins in the blood. In conclusion, diabetes nephropathy is closely related to intestinal flora.

#### 2.2.2 Intestinal microbiota of DM retinopathy

DM retinopathy (DR) is another common microvascular complication of DM, and it is also the main cause of vision loss of working age adults worldwide. The DR prevalence rate of patients with type 2 diabetes (T2DM) increased from 28.8% within 5 years to 77.8% within 15 years (45). According to the International diabetes Federation (IDF), there are currently more than 463 million people with type 2 diabetes in the world, which is expected to increase to 700 million by 2045 (46). The imbalance of intestinal microbiota is also related to the occurrence of DM retinopathy. According to research reports, there are approximately 12 genera of bacteria on the ocular surface, with the main microbial composition being pathogenic bacteria such as rod-shaped bacteria and coagulase negative Staphylococcus, Acinetobacter, and Pseudomonas (47, 48). Some studies have shown that the proportion of Bacteroides and Actinobacteria in patients with DM retinopathy is significantly lower than that in healthy individuals. The proportion of acidophilus, Escherichia and Enterobacteriaceae in the microbiota of patients with DM retinopathy increased significantly (49). Liu et al. (50) pointed out that the intestinal microbial characteristics composed of 25 bacterial families can distinguish DR patients from diabetes patients who ignore omental lesions and healthy control groups (51). Among DR patients, the relative abundance of Pasteurella is the lowest, which can be used as a non-invasive biomarker for DR diagnosis. The plasma TMAO level of DR individuals is significantly higher and positively correlated with the occurrence of DR, indicating that gut microbiota may be involved in the occurrence of DR. These findings support that specific changes in intestinal microbiome and fungal group are associated with DM retinopathy.

#### 2.2.3 Intestinal microbiota of DM neuropathy

DM neuropathy is the result of demyelination, axonal atrophy and inflammation. The main manifestation is distal polyneuropathy (DPN), which occurs in 30-50% of DM patients after long-term exposure to hyperglycemia (43). The intestinal microbiota of patients with DM neuropathy, DM patients without DM neuropathy, and healthy individuals were compared. The results showed that compared with DM patients without DM neuropathy and healthy individuals, DM nephropathy patients had an increase in chlamydia and actinomycetes, and a decrease in bacteroides. In addition, at the genus level, the number of Bacteroidetes and Fecal bacilli decreased, while the number of Shigella, Lachnochostridium, Blautia, Megasphaera, and Rumincoccus torque increased. It is speculated that these changes in the gut microbiota are a result of insulin resistance (11). Xie et al. (52) found that the intestinal flora of stz induced DPN (diabetes neuropathy) rats was dysfunctional, which was mainly manifested by the enrichment of Klebsiella, fecal coccus, Prevotella and other bacterial groups.

In addition, the microbiota of the gut microbiota is involved in regulating human health and metabolism (53). There are numerous experiments supporting the role of gut microbiota in T2DM. Research has shown that taking probiotics to regulate gut microbiota can inhibit inflammatory responses, improve intestinal barrier function, antagonize pathogens, and produce beneficial microbial metabolites, including SCFAs and BAs (54). Research shows that the treatment of Achaematophila mucilaginis can improve the liver function of DM mice, reduce oxidative stress and inhibit inflammation. Lactobacillus mucosum supplementation also improved glucose tolerance and insulin sensitivity in DM mice.

To sum up, the intestinal bacteria of DM patients will produce obvious changes in type and quantity before and after the onset of disease, and the development of DM and its complications can be intervened or improved by regulating the intestinal microbiota.

## 3 Study on the mechanism of TCM intervention on DM by regulating intestinal micromicrobiota

**Figure 2:**
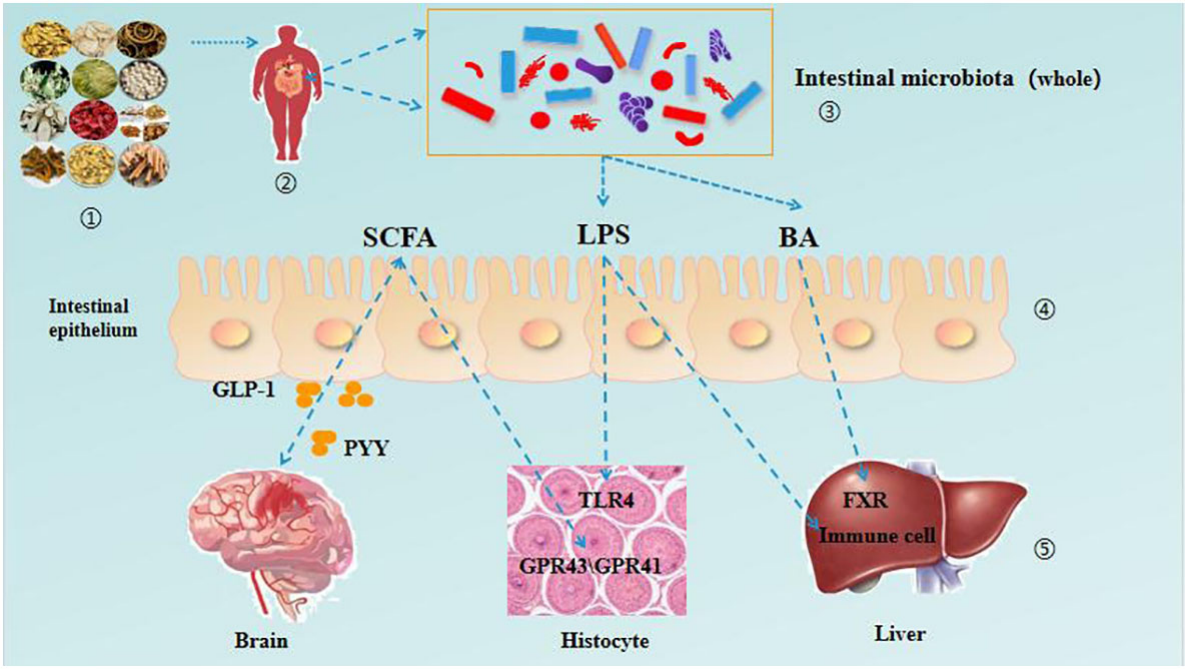
TCM regulates the interaction between intestinal microbiota and various organs in patients with DM.

The pathological mechanism of DM is complex, the course of disease is long and there are many complications. To treat DM, we must grasp the overall dynamics of the disease and treat it according to syndrome differentiation. The gut microbiota not only participates in the body’s energy metabolism, regulates blood sugar and blood lipids, but also regulates the body’s immune system. TCM can intervene and treat the occurrence and development of DM by regulating intestinal microbiota. Research has shown that the rich bioactive components in TCM have a protective effect on the balance of the gut microbiota, directly or indirectly regulating the imbalance of the gut microbiota. Meanwhile, TCM ingredients can regulate the composition and structure of gut microbiota, improve the gut microbiota, and further promote the transformation and utilization of bioactive ingredients in TCM. There is a bidirectional relationship between gut microbiota and TCM (55). In recent years, it has been found that intestinal microbiota and its metabolites are involved in the prevention and treatment of DM through multiple pathways, such as intestinal microbiota short chain fatty acid (SCFA) metabolism, bile acid metabolism, lipopolysaccharide secretion and other pathways. These pathways play a role in the intervention of the occurrence and development of DM, such as maintaining intestinal stability and insulin sensitivity, promoting the absorption and transformation of lipid in the intestinal tract, reducing the inflammatory level induced by endotoxin, regulating blood sugar and restricting diet (56). As shown in **Table 1** and **Figure 2**.

**Table 1:** Research on the mechanism of TCM intervention in DM by regulating intestinal microbiota.

Pathway	Chinese medicine/compound formula	Increase	Reduce	References
**Intestinal microbiota short chain fatty acid (SCFAs) pathway (promoting the production of SCFAs)**	*Scutellaria baicalensis*	*Akkermanisa、Coprococcus、Ruminococcus*	*Odoribacter 、 Parabacteroides*	(49)
	*Ramulus mori*	*Bacteroides、Erysipelotrichaceae*	*Rikenellaceae、Desulfovibrionaceae*	(50)
	*Magnolia officinalis*	*Bacteroidetes*	*Firmicutes*	(55)
	*Highland barley*	*Firmicutes、Actinobacteria、 Verrucomicrobia、Streptococcaceae、Eggerthellaceae、Lachnospiraceae_UCG-006*	*Bacteroidota Proteobacteria、Parasutterella、Helicobacter*	(57)
	*Zuogui Jiangtang Jieyu Fang*	*Firmicutes*	*\*	(58)
	*Resveratrol*	*Bacteroidete、Phascolarctobacterium、Streptococcus、Lachnoclostridium、Parasutterella*	*Firmicutes*	(59)
	*Poria cocos*	*Bacteroidetes*	*Firmicutes*	(60)
**Intestinal microbiota bile acid (BA) pathway (upregulates bile acid synthesis)**	*Huanglian Huangqin*	*Bacteroidales*	*Escherichia-Shigella*	(61)
	*Huanglian Cinnamon*	*Proteobacteria、Verrucomicrobia、Chloroflexi、Akkermansia、Ruminococcus_gnavus_group、Escherichia-Shigella、Parabacteroides*	*Firmicutes*	(62)
	*Huanglian Jiedu Decoction*	*Parabacteroides、* *Blautia、* *Akkermansia*	*Aerococcus, Staphylococcus, Corynebacterium*	(63)
**Intestinal microbiota - inflammatory pathway (reducing inflammation levels)**	*Cornus officinalis*	*Lactobacillus、clostridium*	*staphylococcus*	(64)
	*Ginsenoside*	*Bacteroidetes*	*Firmicutes*	(65)
	*Gegen Qinlian Decoction*	*Faecalibacterium、Bifidobacterium*	*staphylococcus、*	(38)

### 3.1 Intestinal microbiota - short chain fatty acids (SCFAs) pathway

**Figure 3:**
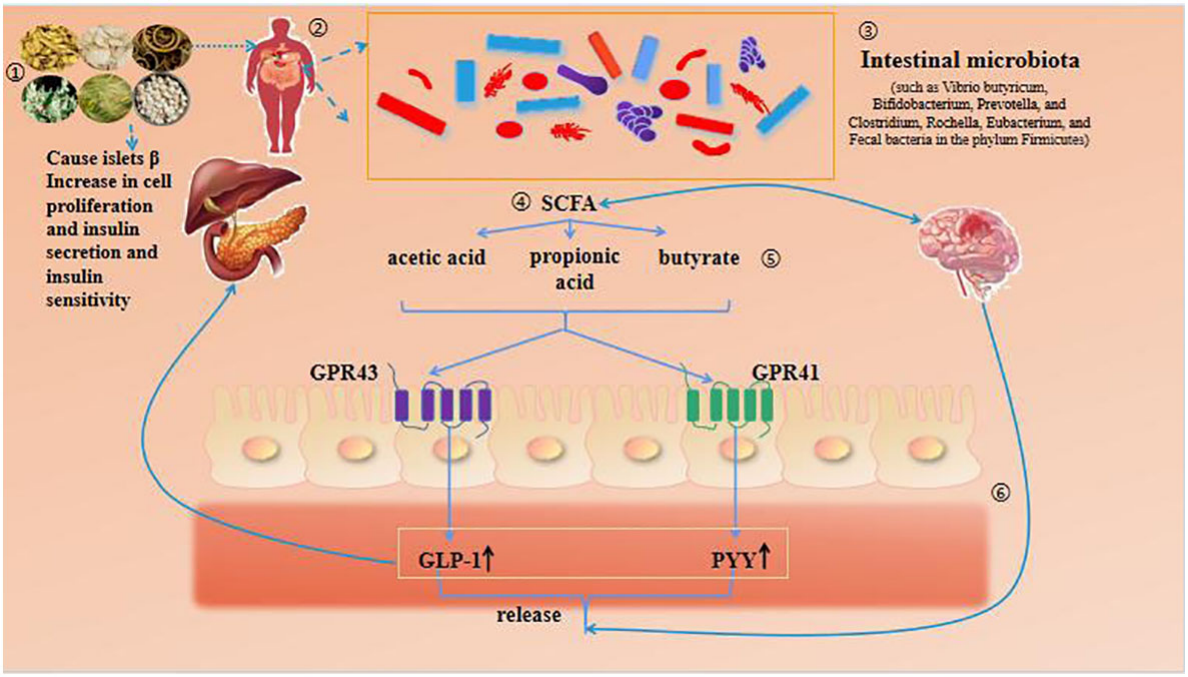
Intestinal microbiota - SCFA (SCFAs) pathway.

Short chain fatty acids (SCFAs) are produced by certain bacteria through fermentation of dietary fiber, with acetic acid, propionic acid, butyric acid, and their salts occupying a dominant position in the human gut (66). SCFA plays a key role in the intervention of intestinal microbiota in DM. As shown in **Figure 3**. On the one hand, SCFAs serve as important energy sources in the body, providing up to 10% of the body’s daily energy needs, directly controlling liver energy synthesis, and maintaining optimal glucose energy levels (57, 67). On the other hand, SCFAs induce the secretion of glucagon like peptide-1 (GLP-1) and gastrointestinal peptide casein YY (PYY) by recognizing and activating G-protein coupled receptor 41 (GPR41) and GPR43 in human cell lines, affecting the systemic effects of glucose metabolism (68). At the same time, the gut microbiota can ferment food that is difficult for the body to digest and absorb to produce short chain fatty acids. SCFAs bind to the G protein coupled receptor Gpr41/Gpr43 in the intestine to improve insulin resistance in the body (58, 69, 70).

It has been found that the gut microbiota associated with SCFA production includes Vibrio butyricum, Bifidobacterium, Prevotella, as well as Clostridium, Rochella, Eubacterium, and Fecal bacteria in the phylum Firmicutes (71, 72). The TCM Scutellaria baicalensis has antibacterial, heat clearing, detoxification, and anti-allergic effects (8). Ju Minzi (73) et al. found that baicalin components in Scutellaria baicalensis improved glucose and lipid metabolism disorders after intervention in high-fat model mice, causing changes in gut microbiota abundance and effectively increasing the amount of short chain fatty acids (acetic acid, propionic acid, and butyric acid) produced by gut microbiota metabolism. Among them, acetic acid was produced the most, corresponding to changes in the microbiota Akkermansia, Ruminococcus, and Paraacteroides, which may be related to improving glucose and lipid metabolism abnormalities caused by high-fat diet. The study on the total alkaloids of mulberry branches (SZ-A), an effective component of TCM mulberry branches, found that (59), long-term administration of SZ-A can regulate the diversity and composition ratio of bacteria in the feces of KKAy mice with T2DM, and increase the proportion of beneficial bacteria/harmful bacteria. Among them, bacteria such as Bacteroides and Erysipelothrichaceae can significantly increase the production of short chain fatty acids, Harmful bacterial species such as Rikenellaceae and Desulfovibrionaceae have significantly decreased; SZ-A can regulate the insulin secretion of KKAy mice indirectly or directly by regulating the intestinal microenvironment to achieve its anti DM effect. Houpo phenol is the main active ingredient of the TCM Houpo, which has many beneficial biological effects, such as antibacterial and anti-inflammatory, neuroprotective function, anti-mutation, antioxidant, anti-tumor, etc. (60, 74, 75). LU et al. (21) found that supplementation of high dose magnolol significantly increased the abundance of Akkermansia and Bacteroides and the concentration of SCFAs in feces of male mice, suggesting that magnolol can improve the symptoms related to DM by increasing the abundance of SCFAs producing bacteria in the intestine. Qingke is a crop of the barley genus in the Poaceae family, mainly distributed in high-altitude and cold regions at an altitude of 4200-4500 meters. It has the characteristics of high protein, low fat, and low sugar. And it is rich in trace elements β- Nutrients such as glucan have been shown to have functions such as regulating blood sugar and improving immunity in barley (22, 76, 77). A large number of researchers (78) attribute the functional activity of barley to its rich content of reased the abundance of Akkerease the concentration of short chain fatty acids (SCFAs) in the cecal contents of T2D mice, including acetic acid, propionic acid, and butyric acid. HBG also has a certain regulatory effect on the gut microbiota of T2D mice, mainly manifested in increasing the abundance of Firmicutes, Actinobacteria, and Verrucomicrobia at the phylum level, while reducing the abundance of Bacteroidota and Proteobacteria. The Zuo Gui blood sugar lowering and depression relieving formula is based on the Zuo Gui Wan in the Jingyue Quanshu (64). Li Wei (61) and other researchers found that the rats with DM complicated with depression were compared with the model group after being treated with Zuogui Jiangtang Jieyu Formula. It significantly increased the abundance of Firmicutes in the intestinal microbiota of rats with DM and depression; And it can significantly increase the content of SCFAs (acetic acid, butyric acid, valeric acid, and isovaleric acid). It proves that Zuogui Jiangtang Jieyu Recipe can effectively improve the intestinal microbiota disorder and regulate SCFAs, thereby improving the glucose metabolism disorder and depression like behavior of DM complicated with depression.

Some studies have shown that the ratio of Firmicutes to Bacteroidetes is considered an indicator of gut microbiota imbalance associated with a high-fat diet (79). Resveratrol belongs to stilbene polyphenols and exists in grapes, mulberries, peanuts and pomegranates (80). *In vitro* and clinical studies have shown that it has a good anti DM effect (62, 81, 82). Li Junye et al. (63) found that resveratrol (RSV) reduced the ratio of Firmicutes/Bacteroidetes in T2DM rats after acting on a high-fat and high sugar diet to establish a T2DM rat model. At the genus level, RSV significantly increased the abundance of Phascolarium, Streptococcus, Lachnoclostridium, and Parasutterella in T2DM rats. Phascolarium and Lachnospiraceae are both bacteria that produce short chain fatty acids (SCFAs) (83). This can prove that RSV increases the abundance of beneficial bacteria in the intestine of T2DM rats and reduces lipid abnormalities in T2DM rats. Poria cocos (Schw.) Wolf, a fungus belonging to the family of porous fungi, is a dried fungal nucleus that has the effects of promoting diuresis and dampness, strengthening the spleen and calming the heart. Its chemical components have various pharmacological effects such as antioxidant, lipid-lowering, and anti-aging (84). Polysaccharides are important components of Poria cocos, with moderate molecular weight and good water solubility. They have pharmacological activities such as anti-tumor, hepatoprotective, anti-inflammatory, and immunomodulatory effects. Duan Yuting (85) found that Poria cocos polysaccharide (PCP) can increase the ratio of Firmicutes/Bacteroidota and the relative abundance of Firmicutes at the phylum level in mice with intestinal mucosal injury. Reduced the relative abundance of Bacteroidota while promoting the production of gut microbiota metabolites SCFAs, resulting in a significant increase in acetic acid and propionic acid content. Therefore, PCP can promote the generation of SCFAs by altering the composition of gut microbiota.

In summary, by increasing the content of acetic acid, butyric acid, valeric acid, and hexanoic acid, the SCFA content in the intestine can be altered, which in turn affects downstream blood glucose and energy metabolism.

### 3.2 Intestinal microbiota - bile acid (BA) pathway

**Figure 4:**
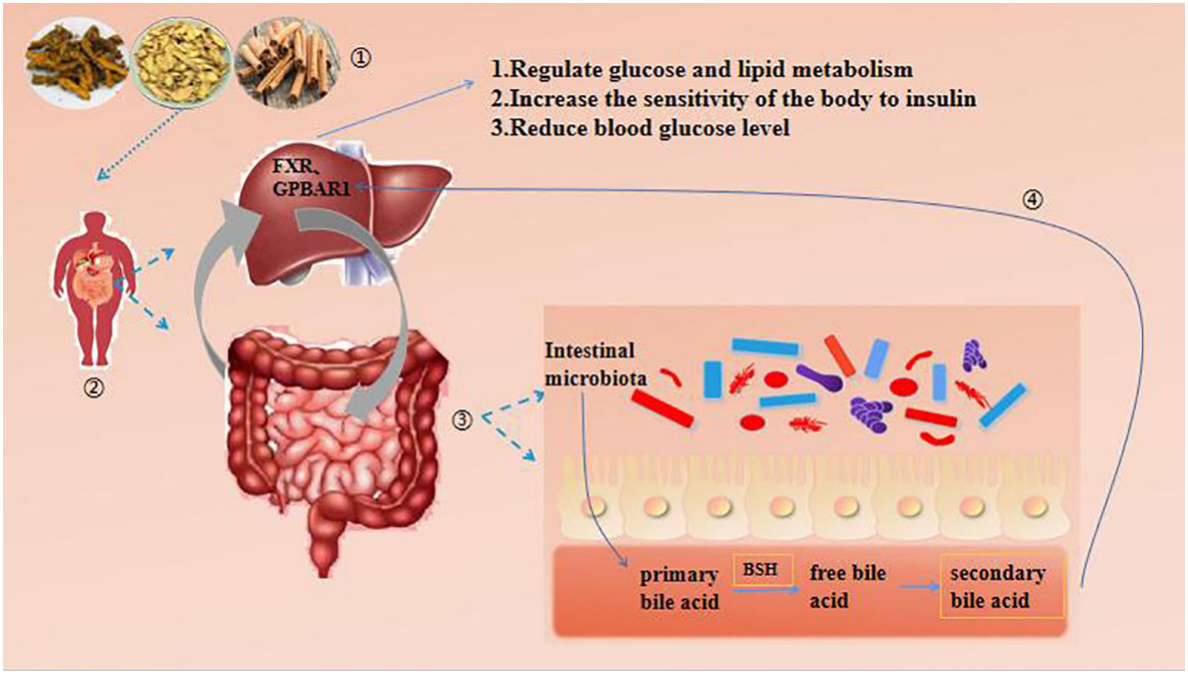
Intestinal microbiota - BA pathway.

Bile acid (BA) is an important component of bile, which is converted from cholesterol by the liver (86). Bile acid can promote the absorption and transformation of lipids in the intestine (87). The intestinal microbiota plays an important role in the synthesis and metabolism of bile acid. The intestinal microbiota will express a bile acid salt hydrolase (BSH), which can cleave the amino acids in the combined bile acid, release the unbound bile acid, and further modify it to form secondary bile acid (88). Bile acids regulate glucose and lipid metabolism, increase insulin sensitivity, and lower blood glucose levels by binding to G protein coupled bile acid receptors (TGR5) and farnesol X receptors (FXR) (65, 89). As the most common antipyretic drug pair, Scutellariae Radix - Coptidis Rhizoma appears in many famous prescriptions in ancient and modern times, such as Gegen Qinlian Decoction, Banxia Xiexin Decoction, Zingiberis Rhizoma, Scutellariae Radix, Coptidis Rhizoma and Ginseng Radix et Rhizoma decoction (90). Modern research shows that the intervention of Scutellariae Radix - Coptidis Rhizoma in T2DM involves the intestinal microbiota - bile acid pathway. Xiao S et al. (91) used Scutellariae Radix - Coptidis Rhizoma to intervene T2DM rats, and found that the abundance of bile acid metabolizing bacteria in the intestinal microbiota of rats decreased, and the level of secondary bile acid decreased, which played a role in preventing and treating T2DM. Coptidis Rhizoma - Cinnamomi Cortex are the components of the classic prescription Jiaotai pills. A number of experimental studies have shown that Coptidis Rhizoma - Cinnamomi Cortex have a good hypoglycemic effect (92–94). Guohua Mu and other researchers found that Coptidis Rhizoma - Cinnamomi Cortex can regulate the secretion of bile acid in spontaneous T2DM db/db mice. Through bile acid, it can affect the synthesis and secretion of TGR5 and GLP-1 in their bodies, reduce their insulin resistance, and then achieve the goal of regulating blood sugar (95). The TCM compound Huanglian Jiedu Decoction was first recorded in Waitai Secret (96) of the Tang Dynasty. Research shows that Huanglian Jiedu Decoction can help correct the disorder of lipid metabolism, alleviate systemic inflammation, reduce fasting blood sugar and improve antioxidant capacity in DM mice. At the same time, it was found that (97) Huanglian Jiedu Decoction intervention in T2DM rats increased the abundance of anti-inflammatory bacteria and regulated dysfunctional gut microbiota, including upregulation of bile acid biosynthesis, reduction of glycolysis, gluconeogenesis, and nucleotide metabolism. It can be seen that gut microbiota plays an important role in bile acid synthesis metabolism. See **Figure 4**.

### 3.3 Intestinal microbiota - inflammatory pathway

**Figure 5:**
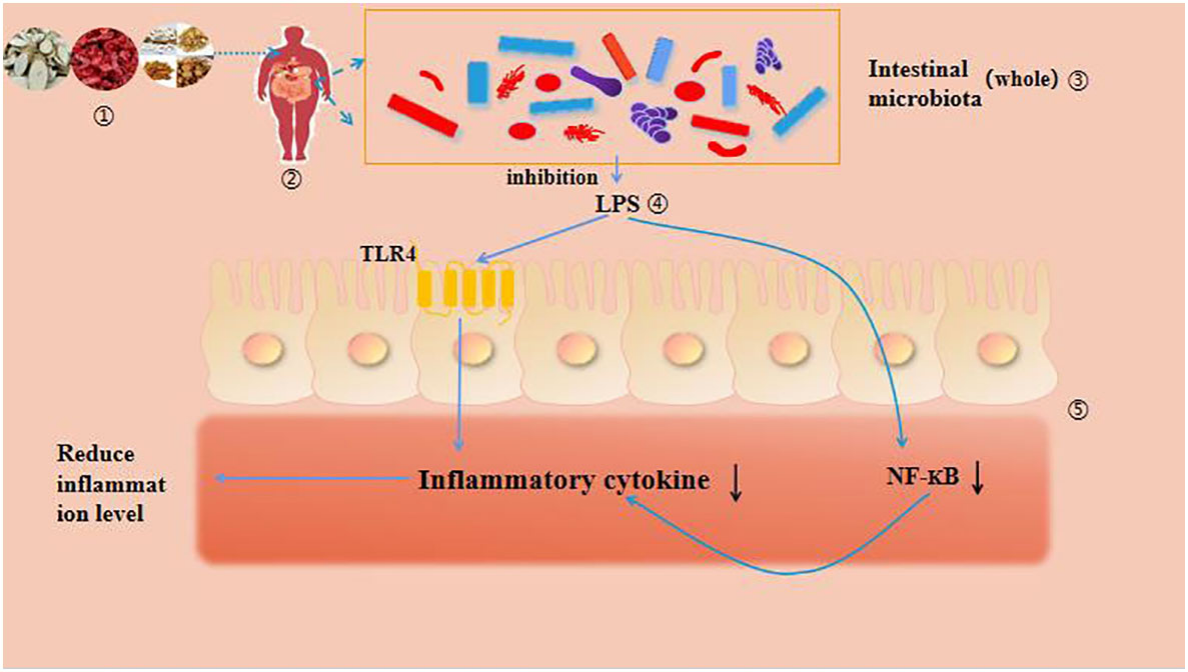
Intestinal microbiota - inflammatory pathway.

LPS, also known as endotoxin, is the main component of the outer membrane of Gram negative bacterial cell walls and an important inflammatory stimulus that can interact with the gut microbiota, ultimately leading to obesity and T2DM (95). As shown in **Figure 5**. Clinical studies have found that T2DM patients are often accompanied by systemic chronic inflammation, mainly due to blood glucose disorders and dysbiosis of the gut microbiota, resulting in damage to the intestinal mucosal barrier and LPS induced inflammation (98). TCM can effectively intervene in the prevention and treatment of T2DM through the gut microbiota LPS pathway. Cornus officinalis contains a variety of effective ingredients, which can protect the liver, treat arrhythmia, regulate immunity, prevent T2DM and its complications (99). Niu D et al. (100) intervened with Cornus officinalis extract in T2DM mice, improving glucose and lipid metabolism disorders and upregulating the secretion of GLP-1 and GLP-2. They also alleviated T2DM symptoms by upregulating the abundance of Gram positive bacteria (Lactobacillus, Clostridium, etc.) and downregulating the abundance of Gram negative bacteria, inhibiting the LPS lipopolysaccharide binding protein (LBP) - monocyte chemotactic protein-1 (MCP-1) - CD14-TLR4 signaling pathway. Ginsenosides have a wide range of pharmacological effects, such as anti DM, anti-inflammatory, liver protection and cardiovascular protection (101–104). Research by Yang Wei et al. (105) has shown that ginsenoside Rg5 not only improves hyperglycemic symptoms in db/db mice, repairs intestinal barrier function, alleviates metabolic endotoxin related inflammation, but also reverses dysbiosis of the colon gut microbiota. By inhibiting the LPS-TLR4 signaling pathway, inflammation in db/db mice is alleviated, thereby alleviating symptoms of T2DM. Ge Gen Qin Lian Decoction is recorded in the Treatise on Cold Damage and Miscellaneous Diseases and has been reported to have a hypoglycemic effect. Some studies have found that after the treatment of Gegen Qinlian Decoction, the relative abundance of Faecalibacterium, Bifidobacterium and other intestinal beneficial bacteria in DM patients has significantly increased, LPS, TNF- s, The level of IL-6 significantly decreased. It is proved that Gegen Qinlian Decoction has the effect of improving the symptoms of DM (106). Based on this, improving the structure of gut microbiota can enhance intestinal barrier function or reduce the abundance of pathogenic bacteria to alleviate endotoxin induced inflammation levels, thereby inhibiting the progression of T2DM (107).

To sum up, a large number of experimental studies have shown that intestinal microbiota disorder is closely related to the occurrence and development of DM, and TCM and compound medicine can achieve the purpose of preventing and treating DM through various ways of intestinal microbiota.

### 3.4 Other treatment mechanisms

Relevant research shows that Chinese medicine can also intervene and treat the development of DM and its complications by regulating the secretion of gastrointestinal hormones, inducing the expression of related genes, etc. DM may be a disorder of gastrointestinal hormone secretion, where gastrointestinal hormones are peptides released into the circulation by endocrine cells and neurons in the gastrointestinal tract (108). It is an important substance that regulates appetite and energy balance, and its physiological functions include regulating blood sugar levels, gastrointestinal peristalsis and growth, and affecting adipocyte function (109). Therefore, changes in the levels and effects of gastrointestinal hormones may play an important role in metabolic diseases such as DM.

The TCM compound Shen Ling Bai Zhu San has the effects of promoting water infiltration, invigorating the spleen and qi, and reducing turbidity and blood lipids. Gao Ping (110) and other researchers found that after the addition of Shenling Baizhu Powder on the basis of metformin treatment, the motilin level of obese T2DMpatients with spleen deficiency and dampness stagnation syndrome was lower than the control group, and the somatostatin level was higher than the control group. Helps to lower blood sugar and blood lipids, thereby helping to suppress the worsening of the condition. Da Huang Gan Cao Tang has the functions of regulating qi and strengthening the spleen, nourishing yin and benefiting the stomach, supplementing qi and nourishing blood. It can help patients reduce stomach qi, restore stomach yin, and harmonize the spleen and stomach. Pan He (111) found that the combination of rhubarb and licorice decoction and acupuncture can improve the level of serum gastric hormone in patients with DM gastroparesis. Significantly reducing MTL and GAS levels, increasing SS gastrointestinal hormone levels, and better promoting gastric emptying, with high reliability. Houpu Qiqi Tang has the effect of promoting qi circulation and regulating qi. Modern medicine believes that it may have pharmacological effects such as correcting gastrointestinal motility disorders and regulating gastrointestinal hormones (112). Huang Peipei et al. (113) found that after treatment with Houpu Qi Qi Qi Tang. The serum gastrin and motilin water of T2DMgastroparesis patients were lower than those of the control group, and the somatostatin level was higher than that of the control group. It is proved that Houpu Exhaust Decoction can effectively alleviate the clinical symptoms of T2DMgastroparesis patients, improve gastric motility, and regulate gastrointestinal hormone levels.

In addition to insulin injection and oral hypoglycemic drugs, gene therapy and other treatment methods began to be applied to the treatment of DM (23). Clinical studies have shown that gene therapy is safe and effective for various complex diseases. Gene therapy for DM can achieve long-term normal blood glucose without exogenous insulin supply (114). Ge Gen Qin Lian Tang (GQD) is a TCM formula used for long-term treatment of common metabolic diseases. Xu X (115) and other studies found that GQD treatment significantly changed the overall intestinal microbiota structure of DM rats. After treatment, the concentration of serum pro-inflammatory cytokines and the expression of immune related genes (including Nfkb1, Stat1, and Ifnrg1) in the pancreas were significantly reduced, thereby alleviating intestinal inflammation and lowering blood sugar.

## 4 Discussion

As a serious disorder of glucose metabolism, DM is not only accompanied by elevated blood sugar, lipid metabolism disorder and systemic inflammation. It can also cause various serious complications, thereby affecting the quality of life of patients. The gut microbiota is established with the development of the host, and its composition is influenced by various factors such as obesity and pregnancy (116, 117). In recent years, research has found that the gut microbiota is closely related to the heat production process of adipose tissue (118). Obesity and overweight caused by abnormal deposition of adipose tissue not only affect nearly 2 billion people worldwide, reduce their quality of life, but also increase the risk of T2DM(T2DM) and several other chronic diseases (119). Adipose tissue, as an endocrine organ and energy storage, is crucial for the overall metabolic homeostasis. Mammalian adipose tissue can be divided into two categories, namely white adipose tissue (WAT) and brown adipose tissue (BAT). The main function of WAT is to store excess energy in the form of triglycerides, while BAT is a thermogenic tissue that plays a crucial role in maintaining core body temperature. White adipocytes have high plasticity and can differentiate into beige adipocytes under stimulation from exercise, cold exposure, and other factors. Beige adipocytes have many morphological and functional characteristics similar to brown adipocytes. This phenomenon is also known as “WAT browning”. The gut microbiota can activate BAT, promote WAT browning, and improve glucose homeostasis. In obesity, the increase in adipocyte size is related to the increased release of pro-inflammatory adipocytokines such as tumor necrosis factor (TNF) and interleukin-6 (IL-6) (120). By forming an inflammatory environment of adipose tissue to address the problem of malnutrition, the resulting inflammatory environment attracts more immune cells to infiltrate, further exacerbating the inflammatory response and inhibiting cellular metabolic function (121). In addition to proteins, adipocytes can also secrete bioactive lipids to regulate systemic metabolism. For example, palmitic acid secreted by adipocytes can improve insulin resistance associated with obesity (122). These observations indicate that adipocytes have multiple roles in different metabolic states. Obesity can lead to structural and functional disorders of cells, and locally activated immune cells produce pro-inflammatory cytokines in response to stress environments. Through various signaling pathways, it gradually evolves into chronic inflammation and insulin resistance. The two will also have a reverse effect on cells, affecting changes at the cellular level. Therefore, changes at the cellular and molecular levels in patients with DM do not exist independently, but will affect each other (123).

TCM focuses on regulating the metabolism of the body, with less toxic and side effects, and is more suitable for the treatment of chronic metabolic diseases such as DM. But currently, the analysis of TCM ingredients is mostly based on single herbs. The chemical components of a TCM compound after co decoction with multiple drugs are not simply the sum of the chemical components of a single drug in the formula, but rather the mutual promotion or inhibition effects between multiple components. Therefore, the complexity and mechanism of action of the components in compound TCM are still difficult to elucidate and have not been widely accepted. In recent years, as new ideas and technologies are constantly proposed abroad to treat DM, researchers have begun to use western medicine combined with Chinese herbal decoction to treat complications of DM, and have achieved remarkable results. The combination of TCM and western medicine provides a new idea for the treatment of DM, and accelerates the research progress in the prevention and treatment of DM (124).But at the same time, there are also many shortcomings, such as the shallow understanding of the pathogenesis of DM complications, and the imperfect experimental design scheme, which need to be constantly improved and perfected in the research. TCM emphasizes the whole in treatment, and the interaction between TCM and gut microbiota is conducive to supplementing and improving the theoretical basis of TCM. Therefore, exploring the effective ingredients of TCM to improve diseases, utilizing the prebiotic ingredients of TCM to improve the composition of the microbiota, and achieving dual treatment of the body and microbiota. Continue to expand the methods of western medicine combined with TCM decoction to treat the complications of DM, so as to provide effective reference for the follow-up people to study and treat the complications of DM. This will be the future development of DM treatment.

## 5 Summary

To sum up, TCM can improve and alleviate the symptoms of DM patients by regulating the imbalance of intestinal microbiota. From the perspective of gut microbiota, this article summarizes and summarizes the pathways of TCM and its compound formulas through gut microbiota short chain fatty acid (SCFA) metabolism, bile acid metabolism, lipopolysaccharide secretion, gut hormone secretion, and induction of gene expression. Maintain intestinal stability and insulin sensitivity, promote the absorption and transformation of lipids in the intestine, reduce endotoxin induced inflammation levels, and regulate blood sugar. TCM and compound Chinese medicine can inhibit the growth of harmful bacteria, promote the growth of probiotics, thus maintaining the balance of intestinal microbiota, and achieve the purpose of preventing and treating DM.

## Author contributions

YP: Writing – original draft, Data curation. JL: Writing – original draft, Conceptualization. LW: Writing – review & editing, Supervision. HQ: Writing – review & editing, Supervision. YZ: Writing – review & editing, Supervision.

## References

[1] Wu Y Ding Y Tanaka Y Zhang W. Risk factors contributing to T2DMand recent advances in the treatment and prevention. Int J Med Sci. (2014) 11:1185–200. doi: 10.7150/ijms.10001 PMC4166864 25249787

[2] Cho N Shaw J Karuranga S Huang Y Rocha Fernandes J Ohlrogge A. IDF DM Atlas: Global estimates of DM prevalence for 2017 and projections for 2045. DM Res Clin Pract. (2018) 138:271–81. doi: 10.1016/j.diabres.2018.02.023 29496507

[3] Ma Q Li Y Li P Wang M Wang J Tang Z. Research progress in the relationship between type 2 DM mellitus and intestinal microbiota. Biomedicine pharmacotherapy = Biomedecine pharmacotherapie. (2019) 117:109138. doi: 10.1016/j.biopha.2019.109138 31247468

[4] You Q Dong Y Zhang L. Pancreas of T2DMpatients β The relationship between cellular function and blood glucose fluctuations. Qingdao Med Health J. (2016) 48:169–71. doi: CNKI:SUN:QDYW.0.2016-03-003

[5] Fang J. To explore the mechanism of Ophiopogon japonicus oligosaccharides against T2DMbased on metabonomics and intestinal microbiota analysis. Guangzhou Univ TCM. (2021). doi: 10.27044/d.cnki.ggzzu.2021.000505

[6] Yue Y. The study of Bupiweixieyinhuoshengyang Decoction based on TMAO intestinal microbiota metabolic pathway to intervene in macroangiopathy of T2DM. Tianjin Univ TCM. (2022) 5–7. doi: 10.27368/d.cnki.gtzyy.2022.000426

[7] Yang L Hu Q. Research progress in the treatment of T2DMwith TCM. Chin J Integrated Traditional Western Med. (2020) 40:1007–11. doi: 10.7661/j.cjim.20200701.076.

[8] Tang S Xu Y. Research progress of TCM in treating DM and its effect on intestinal microbiota. Shizhen TCM. (2018) 29:1434–7. doi: CNKI:SUN:SZGY.0.2018-06-057

[9] Zhang Y. Experimental study of Shenqi compound on regulating intestinal microbiota and metabolites of DM GK rats. Chengdu Univ TCM. (2021) 5–11. doi: 10.26988/d.cnki.gcdzu.2021.000036.

[10] Gomaa EZ. Human gut microbiota/microbiome in health and diseases: a review. AntonieVan Leeuwenhoek. (2020) 113:2019–40. doi: 10.1007/s10482-020-01474-7 33136284

[11] Iatcu C Steen A Covasa M. Gut microbiota and complications of type-2 DM. Nutrients. (2021) 14:166. doi: 10.3390/nu14010166 35011044 PMC8747253

[12] Liu L Zhang J Cheng Y Zhu M Xiao Z Ruan G. Gut microbiota: A new target for T2DM prevention and treatment. Front Endocrinol (Lausanne). (2022) 13:958218. doi: 10.3389/fendo.2022.958218 36034447 PMC9402911

[13] Barko P McMichael M Swanson K Williamset D. The gastrointestinal microbiome: AReview. J Vet Intern Med. (2018) 32:9–25. doi: 10.1111/jvim.14875 29171095 PMC5787212

[14] Wang L Feng Z Xiong D Kong L Zhang A Li G. Chinese society of preventive medicine, microecology branch. Consensus on clinical application of gastrointestinal microecology regulators in China (2016 edition). Chin J Microbiol. (2016) 9:193–206. doi: 10.13381/j.cnki.cjm.201606001

[15] Long C Shao H Luo C Yu R Tan Z. Bacterial diversity in the intestinal mucosa of dysbiosis diarrhea mice treated with qiweibaizhu powder. Gastroenterol Res Pract. (2020) 3):1–8. doi: 10.1155/2020/9420129 PMC7097775 32256567

[16] Demirci M Bahar Tokman H Taner Z Keskin FE Çağatay P Ozturk Bakar Y. Bacteroidetes and Firmicutes levels in gut microbiota and effects of hosts TLR2/TLR4 gene expression levels in adult T1DMpatients in Istanbul, Turkey. J DM its complications. (2020) 34:107449. doi: 10.1016/j.jdiacomp.2019.107449 31677982

[17] He Y. The effect of berberine hydrochloride on gut microbiota and blood endotoxin levels in T2DM rats [D]. Master's thesis Shanxi Med Univ. (2015) 9–17. doi: 10.76666/d.D649807

[18] Huang X Zheng X Zheng Z. Study on intestinal microbiota of patients with type 2 DM. Hebei Med. (2011) 17:1041–3. doi: 10.3969/j.issn.1006-6233.2011.08.019

[19] Ding T Sheng L Song B. To treat type 2 DM, choose western medicine or doctor. Family Life Guide. (2023) 39:65–7.

[20] Sun Y. Study on the effect of Yiqi Bushen Recipe on intestinal microbiota of DM rats [D]. Shandong Univ TCM. (2021) 25–9. doi: 10.27282/d.cnki.gsdzu.2021.000207

[21] Lu Y Fan C Li P Lu Y Chang X Qi K. Short chain fatty acids prevent high-fat-diet-induced obesity in mice by regulating G protein-coupled receptors and gut microbiota. Sci Rep. (2016) 6:37589. doi: 10.1038/srep37589 27892486 PMC5124860

[22] Li Y Cai X Wang X Li H Zhang Z Wu Y. The effect of Chaihu Jialonggu Oyster soup on the intestinal microbiota of patients with T2DMand depression. Chin J Integrated Traditional Western Med. (2023) 43:1433–41. doi: 10.7661/j.cjim.20230517.079

[23] Tan S Wong J Sim Y ,Wong S Elhassan S Tan S Lim G. Type 1 and 2 diabetes mellitus: A review on current treatment approach and gene therapy as potential intervention. Diabetes Metab Syndrome: Clin Res Rev. (2019) 13:364–72. doi: 10.1016/j.dsx.2018.10.008 30641727

[24] Zhou Z Mu Y Wang W Zhu D Ji L Xiao H. Endocrine Metabolism Branch of the Chinese Medical Doctor Association, National Clinical Medical Research Center for Metabolic Diseases Chinese expert consensus on classification and diagnosis of DM Chinese. J DM. (2022) 14:120–39. doi: 10.3760/cma.j.cn115791-20211219-00672

[25] Gharravi A Jafar A Ebrahimi M Mahmodi A Pourhashemi E Haseli N. Current status of stem cell therapy, scaffolds for the treatment of diabetes mellitus. Diabetes Metab Syndrome Clin Res Rev. (2018) 12(6):1133–9. doi: 10.1016/j.dsx.2018.06.021. S1871402118302522-. 30168429

[26] Oresic M Simell S Sysi-Aho M Nanto-Salonen K Seppanen-Laakso T Parikka V. Dysregulation of lipid and amino acid metabolism precedes islet autoimmunity in children who later progress to type 1 DM. J Exp Med. (2008) 205(13):2975–84. doi: 10.1084/jem.20081800 PMC2605239 19075291

[27] Vaarala O Atkinson MA Neu J. The “perfect storm” for type 1 DM: the complex interplay between intestinal microbiota, gut permeability, and mucosal immunity. DM. (2008) 57:2555–62. doi: 10.2337/db08-0331 PMC2551660 18820210

[28] Zhao J Ni Y Lu R Liu J Yu C Zhang W. Research progress in the prevention and treatment of DM with TCM based on intestinal microbiota. J Anhui Univ TCM. (2018) 37:89–92. doi: 10.3969/j.issn.2095-7246.2018.05.025

[29] Shen Y Chen S Wei L Qin J. The relationship between intestinal microbiota and DM. Chin J Microbiol. (2017) 29:357–62. doi: 10.13381/j.cnki.cjm.201703027

[30] Wu W Xiang S Shen F Chen Y Zheng L. Investigation of intestinal microbiota situation of elderly patients with type 2 DM. Chin J Gen Pract. (2014) 12:743–744+767. doi: 10.16766/j.cnki.issn.1674-4152.2014.05.035

[31] Chaohui F Wenli W Bin Z. Research on the intestinal microbiota of the 239 patients in the elderly non-intestinal diseases. Chongqing Med. (2012) 41:2400–1. doi: 10.3969/j.issn.1671-8348.2012.23.022

[32] Biassoni R Di M Squillario M Barla A Piccolo G Ugolotti E. Gut microbiota in T1DM-onset pediatric patients:machine-learning algorithms to classify microorganisms as disease linked. J Clin Endocrinol Metab. (2020) 105:dgaa407. doi: 10.1210/clinem/dgaa407 32692360

[33] Zeng Y Hu Y Wu P Feng X Gu C Guo Y. Analysis of intestinal microbiota in obese people with T2DM. Lab Med. (2016) 31:848–53. doi: 10.3969/j.issn.1673-8640.2016.010.003

[34] Li L Gao Y Zhu K Qu J Liang Y Zhang W. The effect of probiotics on glucose and lipid metabolism and oxidative stress level in overweight DM patients. Chin J Clin Health. (2017) 20:4. doi: 10.3969/J.issn.1672-6790.2017.05.019

[35] Qin J Li Y Cai Z Li S Zhu J. A metagenome-wide association study of gut microbiota in type 2 DM. Nature. (2012) 490:55−60. doi: 10.1038/nature11450 23023125

[36] Delzenne N Cani P Everard A Neyrinck A Bindels L. Gut microorganisms as promising targets for the management of type 2 DM. Diabetologia. (2015) 58:2206–17. doi: 10.1007/s00125-015-3712-7 26224102

[37] Baig M Panchal S. Streptozotocin-Induced DM Mellitus in Neonatal Rats: An Insight into its Applications to Induce Diabetic Complications. Curr DM Rev. (2020) 16:26–39. doi: 10.2174/1573399815666190411115829 30973111

[38] Gross J Azevedo M Silveiro S Canani L Caramori M Zelmanovitz T. Diabetic nephropathy: Diagnosis,prevention, and treatment. DM Care. (2005) 28:164–76. doi: 10.2337/diacare.28.1.164 15616252

[39] Zhou M Li X Liu J Wu Y Tan Z Deng N. Adenine's impact on mice's gut and kidney varies with the dosage administered and relates to intestinal microorganisms and enzyme activities. 3 Biotech. (2024) 14:88. doi: 10.1007/s13205-024-03959-y PMC10884393 38406640

[40] Mahmoodpoor F Saadat Y Barzegari A Ardalan M Vahed S. The impact of gut microbiota on kidney function and pathogenesis. Biomedicine pharmacotherapy = Biomedecine pharmacotherapie. (2017) 93:412–9. doi: 10.1016/j.biopha.2017.06.066 28654798

[41] Vaziri N Yuan J Nazertehrani S Ni Z Liu S. Chronic kidney disease causes disruption of gastric and small intestinal epithelial tight junction. Am J Nephrol. (2013) 38:99–103. doi: 10.1159/000353764 23887095

[42] Koichi K Daisuke S Yoshitomi K Yotaro M Paxton T Naoto S. Gut microbiome-derived phenyl sulfate contributes to albuminuria in diabetic kidney disease. Nat Commun. (2019) 10:1835. doi: 10.1038/s41467-019-09735-4 31015435 PMC6478834

[43] Tanase D Gosav E Neculae E Costea C Ciocoiu M Hurjui L. Role of gut microbiota on onset and progression of microvascular complications of T2DM(T2DM). Nutrients. (2020) 12:3719. doi: 10.3390/NU12123719 33276482 PMC7760723

[44] Salguero M Al-Obaide M Singh R Siepmann T Vasylyeva T. Dysbiosis of gram-negative gut microbiota and the associated serum lipopolysaccharide exacerbates inflammation in type 2 diabetic patients with chronic kidney disease. Exp Ther Med. (2019) 18(5):3461–9. doi: 10.3892/ETM.2019.7943 PMC6777309 31602221

[45] Cui Y Zhang M Zhang L Zhang L Kuang J Zhang G. Prevalence and risk factors for diabetic retinopathy in a cross-sectional population-based study from rural southern China: Dongguan Eye Study. BMJ Open. (2019) 9:e023586. doi: 10.1136/bmjopen-2018-023586 PMC6756414 31530585

[46] Jayasudha R Das T. Gut mycobiomes are altered in people with type 2 diabetes mellitus and diabetic retinopathy. PloS One. (2020) 15:e0243077. doi: 10.1371/journal.pone.0243077 33259537 PMC7707496

[47] Wen X Miao L Deng Y Bible P Hu X Zou Y. The influence of age and sex on ocular surface microbiota in healthy adults. Invest Ophthalmol Vis Sci. (2017) 58:6030–7. doi: 10.1167/iovs.17-22957 29196767

[48] Kugadas A Gadjeva M. Impact of microbiome on ocular health. Ocular Surface. (2016) 14(3):342–9. doi: 10.1016/j.jtos.2016.04.004 PMC5082109 27189865

[49] Das T Jayasudha R Chakravarthy S Prashanthi G Bhargava A Tyagi M. Alterations in the gut bacterial microbiome in people with T2DMmellitus and diabetic retinopathy. Sci Rep. (2021) 11:2738. doi: 10.1038/s41598-021-82538-0 33531650 PMC7854632

[50] Liu W Wang C Xia Y Xia W Liu G Ren C. Elevated plasma trimethylamine-N-oxide levels are associated with diabetic retinopathy. Acta Diabetol. (2021) 58:221–9. doi: 10.1007/s00592-020-01610-9 PMC7889550 33064205

[51] Huang Y Wang Z Ma H Ji S Chen Z Cui Z. Dysbiosis and implication of the gut microbiota in diabetic retinopathy. Front Cell Infect Microbiol. (2021) 11:646348. doi: 10.3389/fcimb.2021.646348 33816351 PMC8017229

[52] Xie J Song W Liang X Zhang Q Shi Y Liu W. Jinmaitong ameliorates diabetic peripheral neuropathy in streptozotocin-induced diabetic rats by modulating gut microbiota and neuregulin 1. Aging. (2020) 12(17):17436–58. doi: 10.18632/aging.103750 PMC7521543 32920546

[53] De G Belzer C Aydin O Levin E Levels J. Aalvink S.Distinct fecal and oral microbiota composition in human type 1 DM, an observational study. PloS One. (2017) 12:e0188475. doi: 10.1371/journal.pone.0188475 29211757 PMC5718513

[54] Sanders M Merenstein D Reid G Gibson G Rastall R. Probiotics and prebiotics in intestinal health and disease: from biology to the clinic. Nat Rev Gastroenterol Hepatol. (2019) 16:605–16. doi: 10.1038/s41575-019-0173-3 31296969

[55] Liu J Fang W Tang Z Dai S Lian C Qiao F. Research overview of the interaction between TCM and intestinal microbiota. Chin J Pharm. (2023) 58:1533–9. doi: 10.11669/cpj.2023.17.001

[56] Zhou Z Ji Y Li J Song Y Ren T. The mechanism of intestinal microbiota in T2DMand the regulatory role of TCM. Med Rev. (2021) 27:3237–43. doi: 10.3969/j.issn.1006-2084.2021.16.022

[57] Cunningham A Stephens J Harris D. Intestinal microbiota and their metabolic contribution to T2DMand obesity. J DM Metab Disord. (2021) 20:1855−1870. doi: 10.1007/S40200-021-00858-4 PMC8630233 34900829

[58] Müller T Finan B Bloom S Alessio D Drucker D Flatt P. Tschp M.Glucagon-like peptide 1 (GLP-1). Mol Metab. (2019) 30:72–130. doi: 10.1016/j.molmet.2019.09.010 31767182 PMC6812410

[59] Liu L Liu Q Li C Huan Y Liu Z Liu Y. Preliminary exploration of the effect and mechanism of innovative hypoglycemic TCM mulberry branch total alkaloids on regulating the intestinal pancreatic axis. Chin J Pharmacol Toxicol. (2019) 33:666–7. doi: CNKI:SUN:YLBS.0.2019-09-039

[60] Saito J Sakai Y Nagase H. *In vitro* anti-mutagenic effect of magnolol against direct and indirect mutagens. Mut Res. (2006) 609:68–73. doi: 10.1016/j.mrgentox.2006.06.021 16884943

[61] Liu Y Li P Yang H Zou M Zhao H Guo H. The mechanism of Zuogui Jiangtang Jieyu Recipe in improving hippocampal synaptic damage caused by N-methyl-D-aspartate receptor overexcitation in rats with DM and depression. Chin Herbal Med. (2023) 54:6323–35. doi: 10.7501/j.issn.0253-2670.2023.19.014

[62] Cheng J You J. Resveratrol inhibited Wnt pathway to improve the cognitive function of diabetic rats. Chin J DM. (2020) 28:379–83. doi: CNKI:SUN:ZGTL.0.2020-05-013

[63] Michno A Gruzewska K Ronowska A. Resevatrol inhibits metabolism and effects blood platelet function in type 2 DM. Nutrients. (2022) 14:1633. doi: 10.3390/nu14081633 35458194 PMC9026466

[64] Liu L Li H Zheng H Chen Q. The regulatory effect of Poria cocos extract on blood glucose and intestinal microbiota in T1DMmice. J Northwest Pharm. (2022) 37:89–94. doi: 10.3969/j.issn.1004-2407.2022.06.015

[65] Swann J Want E Geier F Spagou K Wilson I Sidaway J. Systemic gut microbial modulation of bile acid metabolism in host tissue compartments. Proc Natl Acad Sci U.S.A. (2011) 108:4523–30. doi: 10.1073/pnas.1006734107 PMC3063584 20837534

[66] Venegas D Fuente M Landskron G González M Hermoso M. Short chain fatty acids(SCFAs)-mediated gut epithelial and immune Regulation and its Relevance for inflammatory bowel diseases. Front Immunol. (2019) 10:277. doi: 10.3389/fimmu.2019.00277 30915065 PMC6421268

[67] Arora A Behl T Sehgal A Singh S Bungau S. Unravelling the involvement of gut microbiota in T2DMmellitus. Life Sci. (2021) 273:119311. doi: 10.1016/j.lfs.2021.119311 33662428

[68] Wu J Wang K Wang X Pang Y Jiang C. The role of the gut microbiome and its metabolites in metabolic diseases. Protein Cell. (2021) 12:360−373. doi: 10.1007/s13238-020-00814-7 33346905 PMC8106557

[69] Priyadarshini M Kotlo K Dudeja P Layden B. Role of short chain fatty acid receptors in intestinal physiology and pathophysiology. Compr Physiol. (2018) 8 3):1091–115. doi: 10.1002/cphy.c170050 PMC6058973 29978895

[70] Ramracheya R McCulloch L Clark A Wiggins D Johannessen H Olsen M. PYY-dependent restoration of impaired insulin and glucagon secretion in T2DMfollowing roux-en-y gastric bypass surgery. Cell Rep. (2016) 15:944–50. doi: 10.1016/j.celrep.2016.03.091 PMC5063952 27117413

[71] Cuesta-Zuluaga J Mueller N Corrales-Agudelo V Velásquez-Mejía E Escobar J. Metformin is associated with higher relative abundance of mucin-degrading akkermansia muciniphila and several short-chain fatty acid-producing microbiota in the gut. DM Care. (2017) 40:54–62. doi: 10.2337/dc16-1324 27999002

[72] Chang H. Microbiota or short-chain fatty acids: which regulates DM. Cell Mol Immunol. (2018) 15:88–91. doi: 10.1038/cmi.2017.57 28713163 PMC5811682

[73] Ju M. A study on the mechanism of baicalin improving glucose and lipid metabolism in high-fat diet mice by regulating intestinal microbiota. Southeast Univ. (2019) 12–5.

[74] Park J Lee J Jung E Park Y Park D. *In vitro* antibacterial and anti-inflammatory effects of honokiol and magnolol against Propionibacterium sp. Eur J Pharmacol. (2004) 496:189–95. doi: 10.1016/j.ejphar.2004.05.047 15288590

[75] Lin Y Chen H Ko C Chan M. Neuroprotective activity of honokiol and magnolol in cerebellar granule cell damage. Eur J Pharmacol. (2006) 537:64–9. doi: 10.1016/j.neuropharm.2005.04.009 16631734

[76] Lin S Liu J Chang H Yeh S Lin C Lee W. Magnolol suppresses proliferation of cultured human colon and liver cancer cells by inhibiting DNA synthesis and activating apoptosis. J Cell Biochem. (2002) 84:532–44. doi: 10.1002/jcb.10059 11813258

[77] Kusmiati Dhewantara F. Cholesterol-lowering effect of beta glucan extracted from Saccharomyces cerevisiae in rats. Sci Pharm. (2016) 84:153–65. doi: 10.3797/scipharm.ISP.2015.07 PMC4839553 27110506

[78] Wang B Zhang W Li L Kang Z. Optimization of ultrasonic-assisted extraction process and antioxidant activity of highland barley malt polysaccharides. Appl Chem. (2018) 47:1137–9. doi: 10.16581/j.cnki.issn1671-3206.20180330.018

[79] Li J Yu Y Zhang H. Effects of resveratrol on intestinal microbiota and dyslipidemia in T2DMrats. Chin J Microbiol. (2023) 35:931–5. doi: 10.13381/j.cnki.cjm.202308009

[80] Kuang J Zheng X Zhao A Jia W. Changes in bile acid levels and related treatments in metabolic diseases. Strategy. J Shanghai Jiao Tong Univ. (2019) 39:678–83. doi: 10.3969/j.issn.1674-8115.2019.06.019

[81] Yang Q Zhou T Wang Y. Research progress on the mechanism of action of resveratrol in preventing and treating cerebral infarction. Modern Med Clin. (2023) 38:2374–80. doi: 10.7501/j.issn.1674-5515.2023.09.043

[82] Ali S Abdollahi S Mozaffari-Khosravi H. Effect of resveratrol supplementation on hepatic steatosis and crdiovascular indices in overweight subjects with type 2 DM:a double-blind, randomized controlled trial. BMC Cardiovasc Disord. (2022) 22:212. doi: 10.1186/s12872-022-02637-2 35538431 PMC9088077

[83] Wu G Chen J Hoffmann C Bittinger K Chen Y Keilbaugh S. Linking long-term dietary patterns with gut microbial enterotypes. Science. (2011) 334:105–8. doi: 10.1126/SCIENCE.1208344 PMC3368382 21885731

[84] Liu J. Highland barley β- glucan on the effects of blood glucose and gut microbiota. Heilongjiang Bayi Agric Reclamation Univ. (2023) 2–5. doi: 10.27122/d.cnki.ghlnu.2023.000078

[85] Cheng Y Ding Z Zhang Y Jiang Y Wang L. Research progress on chemical structures and pharmacological activities of Poria cocos polysaccharide and its derivatives. Chin J Chin Mat Med. (2020) 45:4332–40. doi: 10.19540/j.cnki.cjcmm.20200624.601 33164360

[86] Li W Lei S Wang J Liu L Yang H. Effects of Zuogui Jiangtang Jieyu Recipe on intestinal microbiota and short chain fatty acid metabolism in rats with DM complicated with depression. Chin J TCM. (2024) 49:208–15. doi: 10.19540/j.cnki.cjcmm.20230908.401 38403353

[87] Shapiro H Kolodziejczyk A Halstuch D Elinav E. Bile acids in glucose metabolism in health and disease. J Exp Med. (2018) 215:383–96. doi: 10.1084/jem.20171965 PMC5789421 29339445

[88] Joyce S Gahan C. Disease-associated changes in bile acid profiles and links to altered gut microbiota. Dig Dis. (2017) 35:169–77. doi: 10.1159/000450907 28249284

[89] Shan C Qiu N Liu M Zha S Song X Du Z. Effects of diet on bile acid metabolism and insulin resistance in type 2 diabetic rats after roux-en-y gastric bypass. Obes Surg. (2018) 28:3044–53. doi: 10.1007/s11695-018-3264-2 29721762

[90] Lin W. Exploration of the application law of huanglian and huangqin medicinal pairs in the treatise on typhoid and miscellaneous diseases. Shandong J TCM. (2020) 38:1. doi: 10.16295/j.cnki.0257-358x.2020.01.005

[91] Xiao S Liu C Chen M Zou J Zhang Z Cui X. Scutellariae radix and coptidis rhizoma ameliorate glycolipid metabolism of type 2 diabetic rats by modulating gut microbiota and its metabolites. Appl Microbiol Biotech nology. (2020) 104:303–17. doi: 10.1007/s00253-019-10174-w 31758238

[92] Yang S. The application of Jiaotai Pill in DM and network pharmacology analysis of its mechanism. Yichun Univ. (2019) 9–13.

[93] Zhang J Zhang B Duan C. The theoretical basis and application of Jiaotai Pill in the treatment of DM. Medicine. (2016) 11:554–7. doi: 10.3969/j.issn.1673-7202.2016.03.048

[94] Sun Y Yang Y Wang D Jiang H Cui N Su B. Research progress on the chemical composition, pharmacological effects, clinical application, and predictive analysis of quality markers of Jiaotai Pills. Chin J TCM. (2020) 45:2784–91. doi: 10.19540/j.cnki.cjcmm.20200328.201 32627451

[95] Mu G. Clinical application of Huanglian Cinnamomum cassia in the treatment of T2DM and its impact on the BA/TGR5/GLP-1 pathway based on gut microbiota. Beijing Univ TCM. (2021) 7–9. doi: 10.26973/d.cnki.gbjzu.2021.000120

[96] Zhang X Deng Y Shi Q He M Chen B Qiu X. Hypolipidemic effect of the Chinese polyherbal Huanglian Jiedu decoction in type 2 diabetic rats and its possible mechanism. Phytomedicine. (2014) 21:615–23. doi: 10.1016/j.phymed.2013.11.004 24368167

[97] Chen M Liao Z Lu B Wang M Xie Z. Huang-Lian-Jie-Dudecoction ameliorates hyperglycemia and insulin resistant in association with gut microbiota modulation. Front Microbiol. (2018) 8(9):2380. doi: 10.3389/fmicb.2018.02380 PMC6186778 30349514

[98] Zhao Q Wang X Hu Q Zhang R Yin Y. Suppression of TLR4 by miR−448 is involved in diabetic development via regulating macrophage polarization. J Pharm Pharmacol. (2019) 71:806–15. doi: 10.1111/jphp.13048 30536833

[99] Zhang Q. Research on different components of cornus officinalis to improve intestinal microbiota of T2DMmice and its mechanism of action. Shaanxi Normal Univ. (2018) 10–14.

[100] Niu D An S Chen X Bi H Zhang Q Wang T. Corni fructus as a naturalResource can treat T2DMbyRegu lating gut microbiota. Am J Chin Med. (2020) 48:1385–407. doi: 10.1142/S0192415X20500688 32907359

[101] Bai L Gao J Wei F Zhao J Wang D Wei J. Therapeutic potential of ginsenosides as an adjuvant treatment for DM. Front Pharmacol. (2018) 9:423. doi: 10.3389/fphar.2018.00423 29765322 PMC5938666

[102] Kim J Yi Y Kim M Cho J. Role of ginsenosides, the main active components of Panax ginseng, in inflammatory responses and diseases. J Gins Res. (2017) 41:435–43. doi: 10.1016/j.jgr.2016.08.004 PMC5628327 29021688

[103] Wang Z Hu J Yan M Xing J Liu W Li W. Caspase-Mediated anti-Apoptotic effect of ginsenoside rg5, a main rare ginsenoside, on acetaminophen-Induced hepatotoxicity in mice. J Agr Food Chem. (2017) 65:9226–36. doi: 10.1021/acs.jafc.7b03361 28965396

[104] Sun Y Liu Y Chen K. Roles and mechanisms of ginsenoside in cardiovascular diseases: progress and perspectives. Sci China Life Sci. (2016) 59:292–8. doi: 10.1007/s11427-016-5007-8 26798041

[105] Wei Y Yang H Zhu C Deng J Fan D. Hypoglycemic effect of ginsenosideRg5 mediated partly by modulating gut microbiota dysbiosis in diabetic db/db mice. J Agric Food Chem. (2020) 68:5107–17. doi: 10.1021/acs.jafc.0c00605. 32307991

[106] Yang Y Wu X. Research progress in the prevention and treatment of DM by intervention of intestinal microbiota with TCM. Chin J Exp Prescriptions. (2021) 27:219–27. doi: 10.13422/j.cnki.syfjx.20202428

[107] Shen L Ao L Xu H Shi J You D Yu X. Poor short-term glyce-mic control in patients with T2DMimpairs the intestinal mucosal barrier:a prospective, single−center, observational study. BMC endocrine disor ders. (2019) 19:29. doi: 10.1186/s12902-019-0354-7 PMC6408809 30849982

[108] Rehfeld J. Central of gastrointestinal endocrinology. Hormone&Metabolic Res. (2004) 36:735–41. doi: 10.1055/s-2004-826154 15655701

[109] Murphy K Bloom S. Gut horses and the regulation of energy homeostasis. Nature. (2006) 444:854–9. doi: 10.1038/nature05484 17167473

[110] Gao P Tu J. Observation on the effect of modified Shenling Baizhu Powder in the treatment of obesity T2DMwith spleen deficiency and dampness stagnation syndrome and the regulating effect of fat hormone and gastrointestinal hormone in patients. Pract Gynecological Endocrine Electronic J. (2020) 7:164–5. doi: 10.16484/j.cnki.issn2095-8803.2020.23.105

[111] Pan H. Effect of Dahuang Gancao Decoction combined with acupuncture on serum gastrointestinal hormone levels in patients with DM gastroparesis. J Pract DM. (2020) 16(05):31–2.

[112] Yu M Zhang T Zhang D. Clinical efficacy and mechanism of TCM ion introduction combined with knee arthroscopic debridement in the treatment of knee osteoarthritis. Western Med. (2018) 30:1138–42. doi: 10.3969/j.issn.1672-3511.2018.08.010

[113] Huang P. Research on the effect of Houpu Exhausting Decoction on clinical symptoms, gastric motility and gastrointestinal hormone levels of T2DMgastroparesis patients. Reflex Ther Rehabil Med. (2022) 3(01):24–7.

[114] Jaén M Vilà L Elias I Jimenez V Rodó J Maggioni L. Long term efficacy and safety of insulin and glucose gene therapy for DM: 8-Year Follow Up in Dogs. Mol Ther Methods Clin Dev. (2017) 6:1–7. doi: 10.1016/j.omtm.2017.03.008 28626777 PMC5466581

[115] Xu X Gao Z Yang F Yang Y Wang J. Antidiabetic effects of gegen qinlian decoction via the gut microbiota are attributable to its key ingredient berberine. Genomics Proteomics Bioinf. (2020) 18:721–36. doi: 10.1016/j.gpb.2019.09.007 PMC8377040 33359679

[116] Ridaura V Faith J Rey F Cheng J Duncan A Kau A. Gut microbiota from twins discordant for obesity modular metabolism in mice. Science. (2013) 341:1241214. doi: 10.1126/science.1241214 24009397 PMC3829625

[117] Koren O Goodrich J Cullender T Spor A Laitinen K Bckhed H. Host remodelling of the gut microbiome and metabolic changes during preheating. Cell. (2012) 150:470–80. doi: 10.1016/j.cell.2012.07.008 PMC3505857 22863002

[118] Moreno N Fernandez R. The gut microbiota modules both brown of white adipose tissue and the activity of brown adipose tissue. Rev Endocrine Metab Disord. (2019) 20:387–97. doi: 10.1007/s11154-019-09523-x 31776853

[119] Rosen E Spiegelman B. What we talk about when we talk about fat. Cell. (2014) 156:20–44. doi: 10.1016/j.cell.2013.12.012 24439368 PMC3934003

[120] Zhu Q Scherer P. Immunological and endocrine functions of adipose tissue: implications for kidney disease. Nat Rev Nephrol. (2017) 14:105. doi: 10.1038/nrneph.2017.157 29199276

[121] Wang T He C. Pro-inflammatory cytokines: The link is between objectivity and osteoarthritis. Cytokine&Growth Factor Rev. (2018) 44:38–50. doi: 10.1016/j.cytogfr.2018.10.002 30340925

[122] Cao H Gerhold K Mayers J Wiest M Watkins S Hotamisligil G. Identification of a lipokine, a lipid hormone linking adipose tissue to systemic metabolism. Cell. (2008) 134:933–44. doi: 10.1016/j.cell.2008.07.048 PMC2728618 18805087

[123] Pu X Zhang L Bao B. Research progress on the relationship between inflammation mediated obesity and T2DMand the corresponding dietary intervention. Food Industry Sci Technol. (2023) 44:472–9. doi: 10.13386/j.issn1002-0306.2022040111.

[124] Li Y Zhang P He J. Review of clinical progress of western medicine combined with TCM decoction in the treatment of DM complications. DM New World. (2020) 23:196–8. doi: 10.16658/j.cnki.1672-4062.2020.05.196

